# The Ubiquitin-Proteasome System in Huntington's Disease: Are Proteasomes Impaired, Initiators of Disease, or Coming to the Rescue?

**DOI:** 10.1155/2012/837015

**Published:** 2012-09-24

**Authors:** Sabine Schipper-Krom, Katrin Juenemann, Eric A. J. Reits

**Affiliations:** Department of Cell Biology and Histology, Academic Medical Center, University of Amsterdam, Meibergdreef 15, 1105 AZ Amsterdam, The Netherlands

## Abstract

Huntington's disease is a progressive neurodegenerative disease, caused by a polyglutamine expansion in the huntingtin protein. A prominent hallmark of the disease is the presence of intracellular aggregates initiated by N-terminal huntingtin fragments containing the polyglutamine repeat, which recruit components of the ubiquitin-proteasome system. While it is commonly thought that proteasomes are irreversibly sequestered into these aggregates leading to impairment of the ubiquitin-proteasome system, the data on proteasomal impairment in Huntington's disease is contradictory. In addition, it has been suggested that proteasomes are unable to actually cleave polyglutamine sequences *in vitro*, thereby releasing aggregation-prone polyglutamine peptides in cells. Here, we discuss how the proteasome is involved in the various stages of polyglutamine aggregation in Huntington's disease, and how alterations in activity may improve clearance of mutant huntingtin fragments.

## 1. Introduction

### 1.1. Huntington's Disease

Huntington's disease (HD) is one of nine polyglutamine (polyQ) disorders know to date, which are caused by an expansion in the CAG repeat sequence of the encoding DNA that is subsequently translated into a polyQ expansion within the disease-related protein [1, 2]. The presence of a glutamine repeat within proteins is a common feature mainly in transcription factors and may mediate in protein-protein interactions [3, 4]. However, when the polyQ repeat exceeds a length of around 37 glutamines the expansion becomes disease causing [5, 6]. The severity of the disease is correlated with the length of the polyQ expansion, as an increasing repeat length correlates with earlier onset of disease and more severe symptoms [1]. There is strong evidence that the polyQ expansion induces a gain of function since insertion of an expanded CAG sequence in the hypoxanthine phosphoribosyltransferase (HPRT) gene, an HD-unrelated gene which is not involved in any polyQ disorders, induced late-onset neurodegeneration and premature death in a mouse model similar to transgenic HD mouse models [7]. In addition, overexpression of polyQ peptides in transgenic mice caused a neurodegenerative phenotype demonstrating that the polyQ stretch by itself induces toxicity [8]. Still, a loss of wildtype huntingtin (htt) function may also contribute to the disease when considering the broad spectrum of functions which are ascribed to wildtype htt [9]. The htt protein, affected in HD, is an ubiquitously expressed protein which is proposed to be important in embryonal development, transcriptional regulation, axonal, and vesicle transport and has an antiapoptotic function [10]. Although htt is ubiquitously expressed, the earliest neuropathological changes in HD are found in the striatum and cerebral cortex, which are involved in motor control, cognition, and sensory pathways [11]. This leads to a cognitive decline in a progressive manner and manifests in motor dysfunction and severe dementia [12]. Furthermore, HD is characterized by psychiatric and emotional disturbances [13]. The fact that particular brain regions are more affected than others suggests that specific neurons are more vulnerable to htt-induced toxicity probably due to cell-specific gene expression, protein-protein interaction, or posttranslational protein modification [14, 15].

While the exact disease mechanisms behind HD remain elusive, many cellular pathways including transcriptional dysregulation, activation of apoptotic pathways, altered neurotransmitter release, mitochondrial dysfunction, and oxidative stress were found to be affected and therefore subjected to research for therapeutic intervention [16]. An important pathological hallmark of all polyQ disorders is the presence of intracellular protein aggregates, similar as observed in other neurodegenerative disorders like Parkinson's disease and Alzheimer's disease. In the case of HD, aggregates found in human HD postmortem brain are composed of mutant htt (mhtt) N-terminal fragments containing the polyQ stretch [17, 18]. The N-terminal mhtt fragments are highly prone to aggregate in the cell, and accumulating evidence suggests that especially small aggregates of oligomeric mhtt cause cellular toxicity [19–21]. Improving the clearance of these intermediate aggregates or monomeric mhtt fragments should therefore be a therapeutic target to prevent or delay the onset of HD. 

### 1.2. The Ubiquitin-Proteasome System

**Figure 1:**
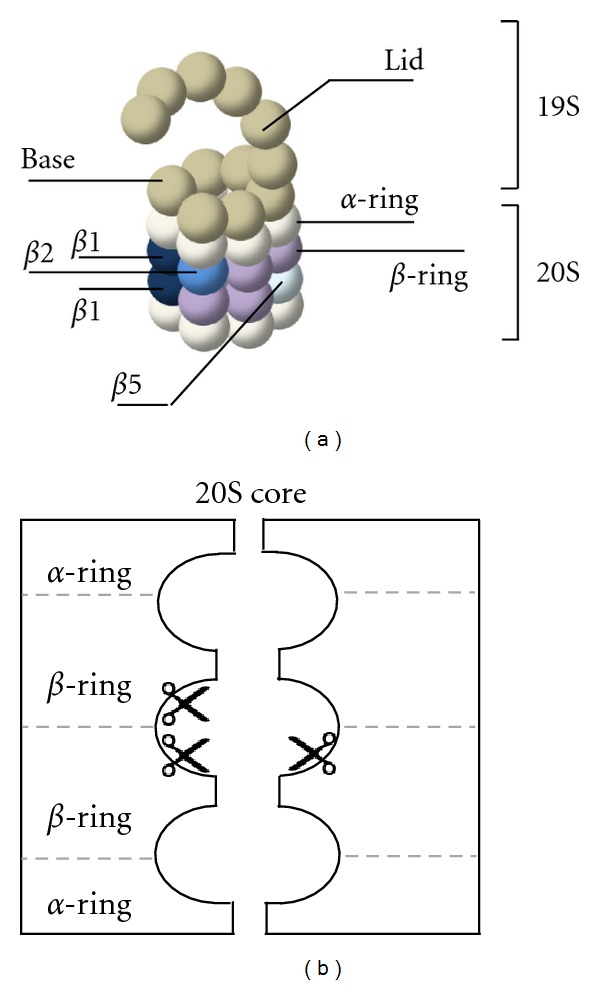
Representation of the 26S proteasome. (a) The 26S proteasome consists of a 20S core capped by one or two 19S activators. The catalytic subunits *β*1, *β*2 and *β*5 are represented in blue in the *β*-ring. (b) A schematic model of the 20S core, indicating the presence of cleavage sites inside the barrel-shaped structure.

The two main pathways involved in the degradation of intracellular proteins are the ubiquitin-proteasome system (UPS) and autophagy. Degradation via the UPS is essential for the clearance of short-lived and misfolded proteins, while autophagy mostly targets long-lived proteins and large structures like protein aggregates or organelles [22, 23]. Both cellular pathways are involved in polyQ protein clearance but at different levels. Degradation of mhtt via macro-autophagy requires targeting of proteins towards lysosomes, which is initiated by engulfment of proteins into autophagosomes. These subsequently fuse with lysosomes to form autolysosomes, resulting in breakdown of their contents by hydrolytic enzymes [24, 25]. However, aggregates of N-terminal mhtt fragments are mainly present in the cell nucleus in human HD postmortem brains [17, 18], while macro-autophagy is a cytoplasmic degradation pathway and therefore not sufficiently effective in clearing nuclear mhtt aggregates. To target nuclear mhtt fragments, the UPS gets into the picture, as proteasomes are present in both the cytoplasm and nucleus. Indeed, various studies indicate that the UPS is involved in processing both wildtype and mhtt [26, 27]. The UPS is mainly involved in maintaining cellular homeostasis via degradation of short-lived regulatory proteins like transcription factors and cell cycle regulatory proteins but also has a protective function since it is responsible for the degradation of damaged and misfolded proteins [28]. Most proteins designated for destruction by the UPS are first tagged by a polyubiquitin chain, which is an ATP-dependent process that occurs via a three steps process. First, Ubiquitin (Ub) is activated by an E1 ubiquitin-activating enzyme, followed by binding to an E2 conjugating enzyme, and finally the binding of the Ub moiety to a lysine residue within the targeted protein via an E3-ligase. Subsequent ubiquitination of the conjugated Ub leads to a poly-Ub chain which designates the protein for targeting towards the 26S proteasome, where the substrates are recognized, unfolded, and degraded [29]. The 26S proteasome includes two major complexes, the 20S core proteasome and the 19S regulatory particle. The 19S regulatory particle recognizes and de-ubiquitinates the polyubiquitinated substrate, unfolds the protein, and guides it through the 20S core [30–32]. The 20S core is a cylindrical complex consisting of four rings stacked on top of each other, while each ring contains seven subunits [33–35] (Figure 1(a)). The two outer rings consist of *α*-subunits that close the interior of the barrel shaped complex, whereas the inner two rings are composed of seven *β*-subunits including three subunits with catalytic activity. These three active subunits, referred to as *β*1, *β*2, and *β*5, have caspase-like activity which cleaves behind acidic residues, trypsin-like activity which cleaves after basic residues, and chymotrypsin-like activity which cleaves behind hydrophobic residues, respectively. When unfolded substrates enter the hollow cavity of the 20S complex, their amino acid chains are then attacked by the N-terminal threonine residue of the catalytic subunits [33, 34, 36, 37] (Figure 1(b)). After cleavage, peptides are released into the cellular environment, where they are further processed by peptidases for antigen presentation or recycled into amino acids.

## 2. The Role of the Proteasome in Huntington's Disease

### 2.1. Proteasomes in HD: The Good, the Bad, or the Ugly?

Various studies indicate that the UPS is involved in processing mhtt since aggregates induced by mhtt are positively stained for Ub and proteasome subunits in human HD postmortem brains, in HD transgenic R6/2 mice that express polyQ-expanded mhtt-exon1(Q145) and in cell culture [17, 38–40]. Also soluble mhtt is polyubiquitinated in cells transfected with mhtt and in HD patient material, suggesting that mhtt can be targeted by the UPS [41–43]. Though, recently it was shown in cell culture that mhtt inclusions are initially devoid of ubiquitin and that soluble mHtt is not extensively ubiquitinated [44]. Furthermore, *in vitro *data suggested that proteasomes may actually be unable to degrade the polyQ repeat present in proteins, as purified mammalian 26S proteasomes were only able to cleave within the flanking sequences or after the first glutamine of a polyQ-containing peptide, while the remaining polyQ repeat was released by the proteasome [45]. One possible consequence of the ineffective degradation of polyQ sequences could be the clogging of proteasomes by long polyQ repeats. Proteasomes generate peptides with an average length of 3–9 amino acids, and these peptides do not exceed a length of 22 amino acids [46]. When confronted with a polyQ-expanded protein, the undigested polyQ peptide is much longer, which may then be unable to diffuse out of the narrow *α*-pore thereby clogging the proteasome, resulting in proteasomal impairment. This hypothesis was supported by FRET experiments showing a stable interaction between the proteasomal catalytic immunosubunit LMP2 and mhtt, although it should be noted that the proteasomal fluorophore was on the outside of the proteasome core, and may thus reflect proteasome binding to htt aggregates [47]. An alternative consequence would be the release of the polyQ peptides generated by the proteasome into the cellular environment, which subsequently would initiate aggregation. Indeed, when mimicking this polyQ peptide release in cells, polyQ peptides exceeding the disease-related threshold of around 40 glutamines showed resistance to degradation, leading to their accumulation and subsequent aggregation [48]. To prevent their accumulation, proteases and peptidases downstream the proteasome should target these polyQ peptides. One of the peptidases shown to be able to target polyQ sequences is puromycin-sensitive aminopeptidase (PSA), which is however, only able to degrade peptides up to 33 amino acids in length [49]. Surprisingly, PSA could still reduce aggregation and toxicity induced by polyQ-expanded peptides or mhtt, but this appears to occur via activation of the autophagy pathway and not via direct degradation by the peptidase [50]. Together this data indicates that proteasomes cannot process polyQ fragments, which consequently could result in proteasome impairment.

Indeed, various studies in cell models and in patient material have reported that the UPS is impaired in HD, which could be the underlying cause of the neurotoxicity. To examine the UPS pathway at different stages of mhtt degradation, a combination of different assays was used to detect alterations in the UPS in striatal cells derived from HttQ111 knock-in mice which express full-length mhtt at endogenous levels [51]. By using small fluorogenic proteasome substrates, as well as various short-lived luciferase reporters which act at different levels of the UPS, it was shown that the UPS is affected at two different levels. A change in activity of the 20S proteasome was detected, as the caspase-like and chymotrypsin-like activities were downregulated, whereas the trypsin-like activity was highly upregulated. Importantly, no effect on the degradation of short-lived proteins that did not require ubiquitination was detected, whereas an increase in the half-life of a polyubiquitinated reporter was observed, indicating that there was a defect in recognition, deubiquitination, or unfolding by the 19S cap. Since an increase in trypsin-like activity was also observed after a stress response upon ATP depletion, it was suggested that expression of mhtt may have caused this change in proteasome activity by a similar, indirect mechanism. In agreement with these results, a decrease in proteasomal caspase-like and chymotrypsin-like activity was also detected both in postmortem brain material and in skin fibroblasts of HD patients, by using small fluorogenic peptides [52]. Together, these studies suggest an overall proteasome impairment both in cells and patient material.

Furthermore, coexpression of mhtt with proteasomal subunits in cells also revealed that recruitment of proteasomes into HD aggregates seems to be irreversible, as fluorescence recovery after photobleaching (FRAP) experiments in living cells using fluorescently-tagged proteasomes showed no recovery of bleached proteasomes that resided in aggregates [47]. These findings led to the conclusion that proteasomes are trapped into polyQ aggregates, which would lead to impairment of the UPS due to Ub and proteasome depletion and even direct blockage or clogging of proteasomes. When using small fluorogenic substrates to quantify proteasome activity, a decrease in proteasomal activity was detected in the soluble fraction of neuronal cells stably expressing an N-terminal fragment of mhtt-Q150, whereas an increased proteasome activity was detected in the insoluble cell fraction containing aggregates [53]. Since there was also a decrease in the degradation of the proteasomal substrate p53, it was concluded that the sequestration of proteasomes into aggregates caused impaired proteasome functionality and neurotoxicity in the cell. This impairment due to sequestration in aggregates was further confirmed by groups using a short-lived GFP^u^ reporter, which has a CL-1 degron signal fused to the C-terminus of GFP, thereby targeting GFP^u^ for proteasomal degradation [54]. When this UPS reporters was cotransfected with polyQ proteins in HEK293 cells, intracellular GFP fluorescence increased 2-3-fold compared to control cells, indicating that proteasome impairment occurred in polyQ protein-expressing cells [55]. The increase in fluorescence and thus proteasome impairment was even higher in polyQ aggregate-containing cells, although it cannot be excluded that this could be due to higher expression levels of the introduced cDNAs in these cells. A global proteasome impairment was reported when mhtt aggregates were present in either the nucleus or the cytoplasm, using GFP^u^ reporters fused to NES or NLS signals to study proteasomal activity in only the cytoplasm or the nucleus of HEK293 cells [56]. While aggregates were present in *trans *compartments, this still led to an increase in GFP fluorescence, suggesting that the UPS was globally affected. Interestingly, this *trans* impairment did not require the presence of mhtt aggregates but also occurred at an earlier stage, indicating that sequestration in aggregates is not a requirement for UPS impairment. Furthermore, this study also showed *in vitro* results which contradict proteasome clogging by mhtt, since purified mhtt aggregates completely failed to impair proteasomes. 

Despite the experiments with purified proteasomes, showing the inability to cleave within polyQ sequences which could lead to proteasomes clogging or continues engagement while trying to degrade mhtt, there are various reports suggesting that proteasomes are capable to digest polyQ sequences. First, proteasomal inhibition increases mhtt levels and in some cases even to a larger extent than macroautophagy inhibitors, although this could also be due to the accumulation of other polyubiquitinated proteins that would co-aggregate and accelerate intracellular aggregation [27]. Secondly, when using degradation signals to target polyQ proteins towards the proteasome less aggregation was observed, indicating that the proteasome can handle polyQ proteins. For example, when an ornithine decarboxylase (ODC) sequence was used to destabilize mhttQ73 in HEK293 cells, an Ub-independent degradation of mhtt was observed, suggesting that the proteolytic activity of the 20S proteasome was not the limiting factor in mhtt degradation [57, 58]. Similarly, when applying the N-end rule to test whether the UPS is capable of unfolding and degrading Ub-R-polyQ-GFP, a complete and efficient degradation of the polyQ protein, without impairment of the proteasome, was shown [59]. Thirdly, when using a NLSsignal to target mhtt to the nucleus (thereby excluding clearance by autophagy), proteasomal degradation of mhtt was facilitated by the nuclear E3-ligase UHRF-2 in stable HeLa cells [42]. This E3-ligase seems to be responsible for ubiquitination of nuclear mhtt and can reduce mhtt aggregation via proteasomal degradation of soluble htt. 

As it appears, proteasome impairment in mhtt-expressing cells remains controversial, and the above mentioned studies showing proteasome impairment have been challenged as well. Using short-lived polyQ-containing proteins that are rapidly targeted for proteasomal degradation via the N-end rule pathway, it was shown that these polyQ proteins were efficiently degraded when targeted towards the proteasome unless these proteins were aggregated [60–62]. Additionally, proteasome activity reporters carrying the N-terminal degron signal but not the polyQ repeat were efficiently degraded in polyQ aggregate-containing cells, implying that proteasomes were still functional in these cells but could not degrade aggregated proteins [60]. Moreover, when examining proteasome activity levels in brains of the conditional HD94 mouse model, which expresses an inducible chimeric mouse/human httQ94^(exon1)^ in the forebrain, the earlier reported UPS impairment could not be detected [63]. But an increase in both the trypsin- and chymotrypsin-like activity was observed, similar to the increase in activities observed in cells expressing so-called immunoproteasomes (induced upon treatment with IFN*γ*, as discussed below). Indeed, labeling for immuno-proteasome subunits confirmed the presence of immuno-proteasomes in brains of HD mice. The absence of proteasome impairment was also more recently underscored in R6/2 mice crossed with transgenic mice expressing different short-lived GFP reporters. Both GFP^u^ and Ub^G76V^-GFP, where the GFP protein is fused to a non-cleavable Ub acting as an Ub-fusion degradation (UFD) signal, have been used as a proteasomal activity marker [61, 64, 65]. In both mouse models, no inhibitory effect by mhtt on proteasomes was detected, contradicting the evidence for proteasome impairment in HD. How to explain these apparently opposite findings in proteasome activity? 

### 2.2. Aggregate Formation Rescues Proteasome Function

As mentioned above, *in vitro *experiments do not show any impairment when proteasomes were incubated with isolated mhtt aggregates and although proteasomes are associated with aggregates, cells still contain a large fraction of proteasomes that are not associated [56, 66]. Together with the observation that proteasomal impairment can already occur before aggregate formation, this argues against a sequestration model. Moreover, a potential protective role was suggested for aggregates when cultured striatal neurons expressing GFP-tagged mhtt-exon1 were visualized by means of an automated fluorescence microscope and followed in time [21]. Surprisingly, neurons that formed large aggregates (called inclusion bodies or IBs) showed a reduction in diffuse mhtt in time and a prolonged survival compared to cells with a diffuse mhtt distribution but no aggregates. When the short-lived UPS reporter, mRFP^u^, was coexpressed to determine proteasome activity in these cells, an improved survival of neurons with IBs was again observed which coincided with less proteasomal impairment [67]. Intriguingly, IB-containing cells showed a significant drop in proteasome activity just before IBs were formed. Together, this suggests that IB formation might be a protective mechanism to sequester toxic mhtt species in the cell that would otherwise impair the UPS. 

Indeed, isolated aggregates do not impair proteasomes *in vitro*, unlike isolated mhtt filaments which induce a reduction in 26S proteasome activity [66]. This suggests that diffuse, oligomeric mhtt can cause proteasomal impairment, whereas IBs do not interfere with the UPS. However, Hipp *et al. *showed that proteasomes do not become clogged *in vitro* by mhtt. This study also excludes in vitro competition between ubiquitinated mhtt and other ubiquitinated proteins for 26S proteasome-dependent degradation [44]. Together, this suggests that mhtt does not directly affect proteasomal activity, but rather maintaining mhtt's solubility will place a burden on the total protein homeostasis machinery. The chaperone network would then become overloaded by aggregation prone mhtt, leading to an overload of proteins that depends on folding and a collapse of the proteolysis network. The observed UPS impairment may therefore reflect the inability of cells to maintain protein homeostasis. This model would be in line with the observed transient accumulation of proteasome reporters in inducible HD94 mice that were crossed with transgenic Ub^-G76V-^GFP mice [68]. Upon expression of the HD94 gene in two-month-old mature mice, a modest increase of the GFP reporter was measured in the first four weeks, indicating a decrease in UPS activity, followed by a decrease in GFP levels when aggregates appeared. When these mice received riluzole (an aggregation preventing compound) an increase in proteasomal reporter levels was again detected. These studies suggest that IB formation is not the bottle neck in progressive neurodegeneration, but that high levels of aggregation-prone mhtt can frustrate the UPS indirectly. 

**Figure 2:**
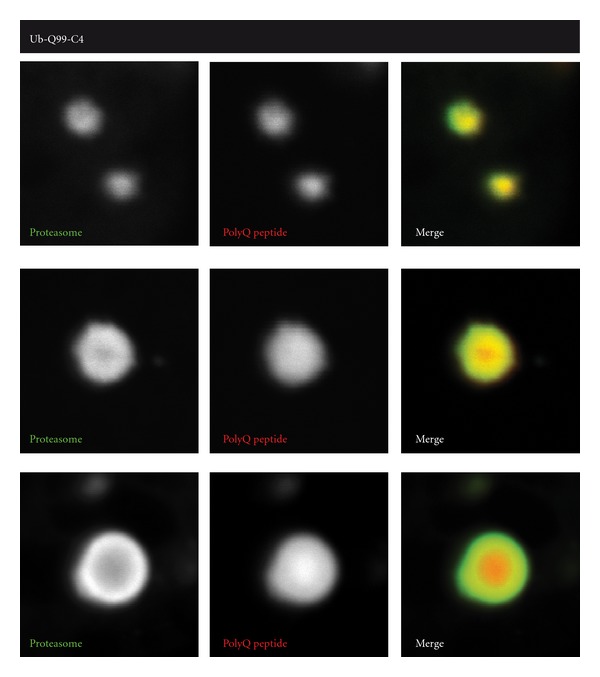
Localization of proteasomes in polyQ aggregates. Upon expression of red-labeled polyQ peptides in Hela cells, initially small aggregates appear (upper panel) which also recruit green-labeled proteasomes. However, in larger polyQ peptide containing aggregates the proteasome is only present in the outer layers, suggesting that proteasomes are not irreversibly trapped in aggregates.

Still, one would expect that the observed sequestration of proteasomes into IBs would affect UPS function. Recent data from our lab suggests that proteasomes are not irreversibly sequestered in mhtt aggregates, but can still move outwards. When fluorescently-tagged proteasomes were co-expressed with fluorescently-tagged polyQ peptides in HeLa cells (green and red, resp., Figure 2), proteasomes were recruited into the polyQ aggregate. While the proteasome is present in the core of the aggregate in newly formed polyQ aggregates (Figure 2, upper panel), the proteasomes only occupy the outskirts of larger aggregates in contrast to the polyQ peptides (Figure 2, lower two panels). This suggests that polyQ fragments but not proteasomes are irreversibly sequestered. This shift to the outside of the larger aggregate probably occurs slowly in time and would explain why no rapid exchange of proteasomes could be observed by photobleaching experiments [47]. Since proteasomes are apparently dynamically recruited to mhtt aggregates, is it then possible to stimulate proteasome activity to improve its capacity to reduce the burden of mhtt?

## 3. Improving Activity of Proteasomes towards PolyQ Proteins

### 3.1. Changing Proteasomal Activity

While purified proteasomes are unable to degrade polyQ repeats, it appears that the UPS in living cells is somehow capable to degrade these polyQ proteins once they are targeted towards the proteasome. This could be due to various modulations in the UPS that occur after specific triggers from the cellular environment, such as alterations in proteasome composition or the recruitment of proteasome activators. If possible, it would be interesting to modify the proteasomal activity to increase cleavage of polyQ sequences? While the constitutive 26S proteasome is comprised of a 20S catalytic core and a 19S activator as described above, the 19S cap can be replaced by the proteasome activator (PA) 28*γ* or the PA28*αβ* activating cap. Furthermore, the 20S catalytic subunits *β*1, *β*2, and *β*5 can be replaced by the immunosubunits LMP2 (PSMB9), LMP7 (PSMB8), and Mecl-1 (PSMB10).

The 20S core has two mechanisms to prevent random cleavage of substrates. First, there is a narrow channel, the *α*-annulus, which closes the catalytic proteasome core to folded proteins (Fig1B) [69]. Secondly, the N-termini of the *α*-subunits form a closed gate which cannot even be entered by small substrates. Thus, for substrates to enter the 20S proteasome, opening of the *α*-gate is necessary. This can be achieved by the 19S cap, which recognizes ubiquitinated proteins, but also by other proteasome activators, jet via a different mechanism. PA28 *α*, *β*, and *γ* are homologous and thus activate the proteasome in a similar fashion. PA28*α* and PA28*β* together form a heteroheptameric ring while PA28*γ* forms a homoheptameric ring [70–72]. These activator rings can dock on the *α*-subunits of the 20S via binding of the PA28 C-termini into the pockets between the *α*-subunits, followed by opening of the proteasome [73, 74]. Unlike the 19S cap, the PA28 caps are ATP-independent and are unable to recognize ubiquitinated and folded proteins, but can stimulate the peptidase activity of the proteasome up to 200-fold dependent on the substrate [74–76]. PA28*αβ* expression is induced upon IFN*γ* stimulation or viral infections, like multiple other genes involved in the immune response, and is therefore proposed to have an important role in antigen processing and presentation [77, 78]. When PA28*αβ* binds the proteasome, all three catalytic activities of the proteasome are increased [79], which is not due to a direct effect on the catalytic subunits, but rather by structural change of the 20S core increasing the accessibility of the catalytic subunits [78, 80]. Furthermore, binding of the PA28*αβ* ring will open the *α*-gate, increasing the uptake but also the release of peptides, which may explain the increase in generated peptides that are more suitable for MHCclassI binding [81]. The function of PA28*γ* in the cell is more diverse, as multiple interaction partners and degradation targets have been identified confirming a role in various cellular processes including cell cycle regulation and apoptosis, both in a 20S-dependent or- independent manner [82, 83]. Proteasome activation by PA28*γ* mainly increases trypsin-like activity, suggesting a conformational change in the 20S core that covers the chymotrypsin-like and caspase-like site and exposes the trypsin-like site [79, 84, 85]. Despite the peptidase activity 20S-PA28*γ* proteasomes are able to cleave intact proteins via unstructured or linker regions [84, 86].

### 3.2. The Proteasome Activator PA28*αβ*

**Figure 3:**
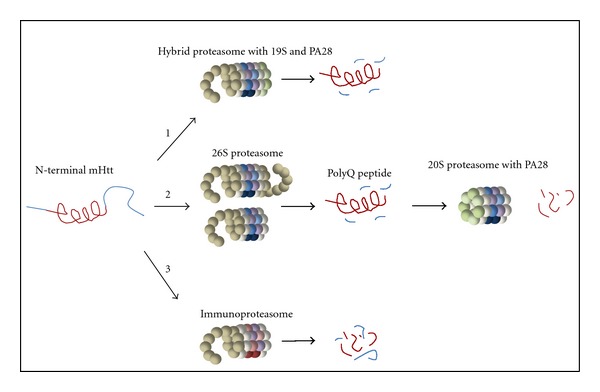
Potential proteasomal pathways for polyQ degradation. (1). Hybrid-proteasomes can recognize ubiquitinated proteins by the 19S cap and release the polyQ peptides faster due to an open gate conformation facilitated by PA28. (2) 26S proteasomes may be able to degrade mhtt but not the actual polyQ tracts. The released polyQ peptide could be targeted by PA28-capped proteasomes. (3) Changing the catalytic activity by immuno-subunit replacement might improve degradation preference for polyQ sequences.

Expression of the proteasome activator PA28*αβ* in patient material increased UPS function in control cells but not in HD fibroblasts, suggesting that introduction of PA28*αβ* would not improve polyQ degradation in mhtt expressing cells [52]. However, these experiments were performed in cells already expressing mhtt, and it would be interesting to induce PA28*αβ* at an earlier stage prior to disease onset in order to study the direct effect of PA28*αβ* on polyQ degradation. Interestingly, PA28*αβ* activation of purified 20S proteasomes increased degradation of short Q-peptides consisting of 10glutamines, and degradation of short peptides with a glutamine at position P1 was increased with PA28*αβ* present [45, 87]. The expression of PA28*αβ* could improve polyQ degradation via two potential pathways, either by so-called hybrid proteasomes or via a two-step, sequential cleavage pathway (Figure 3). Hybrid proteasomes are composed of a 20S particle capped on one side by the 19S complex and on the other side by PA28*αβ* [88]. Here, the 19S cap would recognize and unfold mhtt fragments, whereas PA28*αβ* would operate as an exit channel for generated polyQ peptides, thereby preventing internal clogging of proteasomes (Figure 3, route 1). Although polyQ degradation is not improved, by flushing the polyQ peptides, the proteasome would at least remain active. The second possibility would be a sequential pathway involving two different composed proteasomes (Figure 3, route 2). When indeed the 26S proteasome would be unable to cleave the polyQ sequences present in mhtt, it would release the resulting pure polyQ fragments into the cellular environment [45]. However, PA28*αβ* could bind to both sites of downstream 20S particles thereby opening both gates and enhance the proteasome activities towards polyQ peptide degradation [89]. The frequency of these PA28*αβ* capped proteasomes seems to be low since it was shown that only 4% of the total proteasome pool in rabbit spleen had this composition [90], although this number differs dependent on the cell type used (e.g., 15% in HeLa cells) and can be increased by IFN*γ* [91]. To our knowledge, it is unknown whether these double-capped PA28*αβ* proteasomes are present in brain tissues and whether they are increased during HD.

### 3.3. The Proteasome Activator PA28*γ*

While PA28*αβ* is mainly present in immune-related cells and generally absent from the brain, PA28*γ* could be a better candidate for proteasome activation due to its high expression in neurons [92]. Furthermore, the nuclear localization of PA28*γ*, in contrast to PA28*αβ* which is mainly present in the cytoplasm, makes it an interesting proteasomal activator to target intranuclear mhtt. However, since expression of PA28*γ* inhibits the chymotrypsin-like activity, which seems to be the catalytic site important for cleaving polyQ peptides, it was speculated that downregulation of PA28*γ* would reduce the disease phenotype [65, 85, 93]. When R6/2 mice were crossed with PA28*γ* KO mice, no difference was seen in the behavioral phenotype nor in aggregate formation. In contrast, expression of PA28*γ* showed a protective role in an HD cellular model, but the observed increase in viability of cells that were exposed to stress conditions could also be due to the role of PA28*γ* as an antiapoptotic factor [94, 95]. Additionally, an intriguing mutation at lysine 188 in PA28*γ* altered the activation of the 20S proteasome due to destabilization of the PA28*γ* ring structure [85]. It is thought that due to this unstable conformation, the 20S core is differently structured thereby exposing all active sites with an increase of the proteasome activities similar to the activation changes induced by PA28*αβ*. *In vitro*, it has been demonstrated that the mutated activator PA28*γ* (K188E) increased activation towards polyQ fragments, since Q_10_-peptides were degraded into fragments ranging between 1–9 glutamines [93]. This is in contrast to the earlier studies by Venkatraman and colleagues where it was shown that proteasomes could not cleave polyQ sequence [45]. Varying experimental conditions could explain these differences. As proposed for the PA28*αβ*, also PA28*γ* (K188E) could improve polyQ degradation in two different pathways, either as hybrid proteasomes or by improving cleavage of polyQ peptides released by upstream 26S proteasomes (Figure 3).

### 3.4. Proteasome Immunosubunits

Besides inducing PA28*αβ*, IFN*γ* also induces expression of the proteasome immunosubunits LMP2 (*β*1i), LMP7 (*β*5i), and MECL-1 (*β*2i) which replace the constitutive catalytic subunits *β*1, *β*5, and *β*2, respectively. Incorporation of these newly synthesized subunits happens in de novo formed proteasomes within a time span that is four times faster than assembly of constitutive proteasomes [96, 97]. More important is the induced change in proteasome activity, as replacement of the constitutive subunits by immuno-subunits leads to down-regulation of the caspase-like activity and upregulation of the trypsin-like and chymotrypsin-like activities [98–100], although some discrepancies have been published on the induced alterations in proteasome activity and studies on activity changes induced by individual immuno-subunits also do not give conclusive results [76, 101, 102]. Interestingly, a similar increase in trypsin- and chymotrypsin-like activity of the proteasome is observed in human HD postmortem brains and in the HD94 mouse model, suggesting that immuno-proteasomes are induced in HD [63]. It is tempting to believe that this may reflect a protective response in order to degrade the accumulating polyQ fragments, especially since immuno-proteasomes also appear to be important for cellular homeostasis [103, 104]. Another described consequence of IFN*γ* production is increased protein translation via the mTOR pathway, resulting in the generation of defective, unfolded, and oxidized proteins [105–107]. These defective ribosomal products (DRiPs) become polyubiquitinated and tend to form aggresome-like-induced structures (ALIS) as a cellular response to misfolded protein fragments in the cell. Therefore, simply inducing immuno-proteasomes by IFN*γ* to improve polyQ degradation might be counterintuitive, as the increase in DRIPs would only accelerate aggregation. However, in time the 19S cap dissociates from the 20S core and starts to forms complexes with the newly formed immuno-proteasome 20S particles [104]. These newly formed complexes appear to be better capable in preventing protein accumulations since mice deficient in immuno-subunits showed higher amounts of ALIS after IFN*γ* induction, indicating that immuno-proteasomes may be preferred to deal with the clearance of “dangerous” proteins and fragments. This is further supported by data showing that immuno-proteasomes and PA28*αβ* are also involved in the increased degradation of oxidized proteins after treatment of cells with hydrogen peroxide (H_2_O_2_) [103]. The remaining question is whether we can use these immuno-proteasomes in order to clean up the polyQ fragments that induce toxicity in HD (Figure 3, route 3). The observed presence of immuno-subunits in HD94 mice has been shown to be a secondary effect due to inflammation [63, 108]. However, it remains unknown what would happen if immuno-proteasomes were present at an earlier stage of the disease.

## 4. Concluding Remarks

Since mhtt aggregates are mainly found in the nuclei of the affected neurons of human HD postmortem brain, it seems favorable to increase the degradation capacities in the nucleus. The UPS appears to be a robust mechanism in polyQ expressing cells, as it can recover after a temporary impairment [109]. Although it is clear that the proteasome is involved in the degradation of mhtt, the role of proteasomes remains contradictory. It is unknown whether proteasomes are the good guys as they can efficiently degrade nuclear mhtt fragments, or the bad guys for generating toxic, aggregation-prone polyQ peptides, or even the ugly guys when they become clogged and impaired by the polyQ fragments. In all cases, the modification of proteasome activity could stimulate them to improve clearance and prevent aggregation and toxicity of the polyQ fragments, not only in HD but also in related polyQ disorders. Introduction of different activators, exchanging the catalytic subunits or even using specific proteasome compounds that modulate proteasome activity might lead to improvement of the proteolytic cleavage of polyQ proteins. As proteins with an expanded polyQ stretch need to be soluble to enter the 20S core, a combination of proteasome activation and aggregate preventing compounds or chaperones could benefit the degradation process. It is known that particular heat-shock proteins can decrease aggregation rates in polyQ models, especially two members of the Hsp40 family (DNAJB6 and DNAJB8) are promising candidates [110–113]. Alternatively, chemical compounds and aggregation-interfering peptides like QBP1 could increase the solubility of mhtt to optimize its targeting for proteasomal degradation [20, 114]. Thus far, the general idea is that proteasomes are negatively affected in HD and have a great contribution to the disease course. The actual situation may be less grim, since recent data suggest that proteasomes are not impaired which makes it an interesting therapeutic target.
